# Clinical evaluation of integrated panel testing by next-generation sequencing for somatic mutations in neuroblastomas with *MYCN* unamplification

**DOI:** 10.18632/oncotarget.17917

**Published:** 2017-05-17

**Authors:** Yanna Cao, Yan Jin, Jinpu Yu, Jingfu Wang, Yanli Qiu, Xiaofeng Duan, Yingnan Ye, Yanan Cheng, Li Dong, Xiaolong Feng, Daowei Wang, Zhongyuan Li, Xiangdong Tian, Huijuan Wang, Jie Yan, Qiang Zhao

**Affiliations:** ^1^ Department of Pediatric Oncology, Tianjin Medical University Cancer Institute and Hospital, National Clinical Research Center for Cancer, Key Laboratory of Cancer Prevention and Therapy of Tianjin, Tianjin's Clinical Research Center for Cancer, Tianjin, 300060, P.R. China; ^2^ Department of Cancer Molecular Diagnostic Center, Tianjin Medical University Cancer Institute and Hospital, National Clinical Research Center for Cancer, Key Laboratory of Cancer Prevention and Therapy of Tianjin, Tianjin's Clinical Research Center for Cancer, Tianjin, 300060, P.R. China; ^3^ Key Laboratory of Breast Cancer Prevention and Therapy, Ministry of Education, Tianjin Medical University Cancer Institute and Hospital, National Clinical Research Center for Cancer, Key Laboratory of Cancer Prevention and Therapy, Tianjin, 300060, P.R. China

**Keywords:** neuroblastoma, DNA copy number variations, high-throughput nucleotide sequencing, prognosis, treatment

## Abstract

Neuroblastomas (NBs) exhibit heterogeneity and show clinically significant prognosis classified by genetic alterations. Among prognostic genes or genome factors, *MYCN* amplification (MNA) is the most established genomic marker of poor prognosis in patients with NB. However, the prognostic classification of more than 60% of patients without MNA has yet to be clarified. In this study, the application of target next-generation sequencing (NGS) was extended on the basis of a comprehensive panel of regions where copy number variations (CNVs) or point mutations occurred to improve the prognostic evaluation of these patients and obtain the sequence of 33 patients without MNA. A mean coverage depth of 887× was determined in the target regions in all of the samples, and the mapped read percentage was more than 99%. Somatic mutations in patients without MNA could be precisely defined on the basis of these findings, and 17 unique somatic aberrations, including 14 genes, were identified in 11 patients. Among these variations, most were CNVs with a number of 13. The 3-year event-free survival (EFS) of CNV(−) patients was 60.0% compared with the EFS (16.7%) of CNV(+) patients (*P* = 0.015, HR = 0.1344, 95%, CI = 0.027 to 0.678). CNVs were also associated with unfavorable histological characteristics (*P* = 0.003) and likely to occur in stage 4 (*P* = 0.041). These results might further indicate the role of CNVs in NB chemotherapy resistance (*P* = 0.059) and show CNVs as a therapeutic target. In multivariate analysis, the presence of CNVs was a clinically negative prognostic marker that impaired the outcome of patients without MNA and associated with poor prognosis in this tumor subset. Comprehensive genetic/genomic profiling instead of focusing on single genetic marker should be performed through in-depth NGS that could reveal prognostic information, improve NB target therapy, and provide a basis for investigations on NB pathogenesis.

## INTRODUCTION

Neuroblastoma (NB) is a common extracranial pediatric solid tumor in the sympathetic nervous system, and its incidence rate is approximately 10.54 cases per one million per year in children younger than 15 years [1]. The most important clinical hallmark of NB is biological heterogeneity, and this hallmark is represented by its wide range of clinical behaviors and diverse treatment responses [2], which are closely related to their individual genetic or molecular features. Therefore, NB patients should be subjected to reasonable and accurate stratification depending on genetic or molecular features to improve treatment and prognosis. Studies on Children's Oncology Group (COG) NB have focused on defining the risk groups for patient stratification and protocol assignment based on prognostic factors [3], including age at diagnosis [4], International Neuroblastoma Staging System (INSS) [5–7], International Neuroblastoma Pathology Classification (INPC), *MYCN* status, DNA index [8], 1p loss of heterogeneity (LOH) [9], and 11q LOH [10]. Despite these estimation systems, stratification is poorly implemented and thus results in overtreatment or undertreatment of patients with NB. Among these prognostic genes or genomes, *MYCN* amplification (MNA), associated with rapid disease progression in patients of all ages and stages, is the most characterized and unfavorable prognostic biomarker of NB [4, 11, 12]. However, MNA accounts for approximately 20% of genetic modifications in NB, and over 60% of patients with high-risk NB lack MNA [13]. The markers of these tumors, especially those in children with stage 4 diseases and poor prognosis, are weak. Thus, key factors related to NB should be identified to help effectively manage this disease.

High-risk aggressive tumors are typically characterized by various genomic alterations, including point mutations and copy number variations (CNVs) [14, 15]. Compared with adult tumors, mutation frequencies of tumor cells of children is relatively lower. Somatic chromosomal imbalance variation is a relevant feature of poor prognosis of NB [10, 16–18]. CNVs, amplifications, or deletions of genes of more than 1 kb are classified as chromosomal imbalance variation. In addition to MNA, the CNV of other single gene yields a low occurrence rate. As such, its importance is often disregarded, but the frequency of recurrent CNVs is relatively higher than that of point mutations [19, 20]. Thus far, none of the published CNV-based classifiers, except *MYCN* and 1p and 11q LOH, have been incorporated in clinical classification systems. Therefore, the relevance of CNVs in NB should be further investigated.

Different analytical techniques, including fluorescence *in situ* hybridization (FISH) and comparative genomic hybridization microarrays, have been used to detect CNVs. Next-generation sequencing (NGS) has been developed for clinical applications and future CNV assessment. One study compared the genomic profiles generated through exome sequencing data with those obtained from high-resolution Affymetrix single nucleotide polymorphism (SNP) microarrays and confirmed that several smaller genes can be identified through exome profiling but not through SNP microarray analyses [21]. They concluded that exome sequencing is a useful diagnostic tool to detect CNVs in NBs. Therefore, target-capture NGS was suitable for this study, and its application was extended on the basis of a comprehensive panel of regions where CNVs and point mutations occurred to identify important CNVs other than MNA and to improve the prognostic evaluation of patients without MNA.

## RESULTS

### Patient characteristics

A total of 96 patients were included and evaluated through FISH: 14 MNA(+) patients (4 in non-stage 4 and 10 in stage 4) and 82 MNA(−) patients (31 in non-stage 4 and 51 in stage 4). The three-year event-free survival (EFS) rates of 10 MNA(+) patients and 51 MNA(−) patients in stage 4 were 21% and 47%, respectively. These results indicated that 83.6% of the patients in stage 4 lacked MNA, and their prognosis remained poor. As such, these patients should be examined with other molecular tests to enhance stratification. Finally, 33 patients were evaluated (Supplementary Table 1). The average age of these patients was 38.546 ± 26.126 months, and of these patients, 12 aged ≤ 18 months and 21 aged > 18 months. Of the 33 patients, 16 were males and 17 were females. Histological classification revealed 16 exhibited favorable histology (FH) and 17 manifested unfavorable histology (UH). A total of 54.5% (18/33) patients suffered from distant metastasis, and the mean of serum NSE was 324.700 ± 391.227 ng/mL. Furthermore, 16 patients were in stage 4 (Table 1), 15 patients aged > 18 months, 13 patients were in the UH group, none showed MNA, and all of these patients received similar treatments.

**Table 1:** Clinical data and follow-up results of 16 patients with stage 4 NB

Patient NO.	Age (month) /Age group	INPC	INSS	CNV	Event/ time of occurrence (month)	VGPR
2	72 (2)	UH	4	+	progress/15	NO
6	53 (2)	UH	4	+	progress /16	NO
8	40 (2)	UH	4	−	progress /18	NO
10	27 (2)	UH	4	+	none/35	YES
11	12 (1)	UH	4	+	death/17	NO
15	47 (2)	UH	4	−	none/19	YES
16	81 (2)	UH	4	−	progress /20	YES
17	71 (2)	UH	4	−	none/22	YES
19	67 (2)	UH	4	−	none/19	YES
20	57 (2)	FH	4	−	none/23	YES
22	39 (2)	UH	4	−	relapse /18	NO
26	52 (2)	UH	4	−	none/17	YES
30	62 (2)	UH	4	+	progress/13	NO
31	40 (2)	FH	4	−	progress/17	NO
32	42 (2)	FH	4	−	none/17	YES
33	36 (2)	UH	4	+	relapse /10	NO

### Panel design and NGS data quality assessment

Whole genome sequencing analysis revealed that our panel, which was mainly based on CNVs, covered most of the chromosomal regions and candidate genes associated with NB. The whole genome data of more than 200 NB patients in foreign countries were analyzed and CADD, PROVEAN, and mutation assessor were utilized to filter mutant genes, which were defined as benign mutations. Some of the major CNV regions closely related to NB prognosis were selected because these regions were involved in the deletions or amplifications of genomic chromosomal imbalance and influential to the adverse prognosis of NB. Proto-oncogenes or tumor suppressor genes were also mainly located in these areas. Overall, our NB panel comprised 53 genes and 3 large chromosome regions (Supplementary Table 2). Moreover, 33 biopsies and matched leukocyte samples were subjected to capture-based deep sequencing with our panel to determine and quantify somatic mutations. The detailed quality control data of these samples are shown in Supplementary Table 3. All of the 33 tissue and leukocyte samples sequenced were then subjected to stringent QC allegations. The mean coverage depth in all of the target regions in all of the samples was 887×, and the mapped read percentage was over 99%. The imputed insert size and library complexity statistics shown in Supplementary Figure 1 revealed a mean insert size of 212 bp, and these findings indicated a high capture efficiency of the probes.

### Mutation spectrum

**Figure 1:**
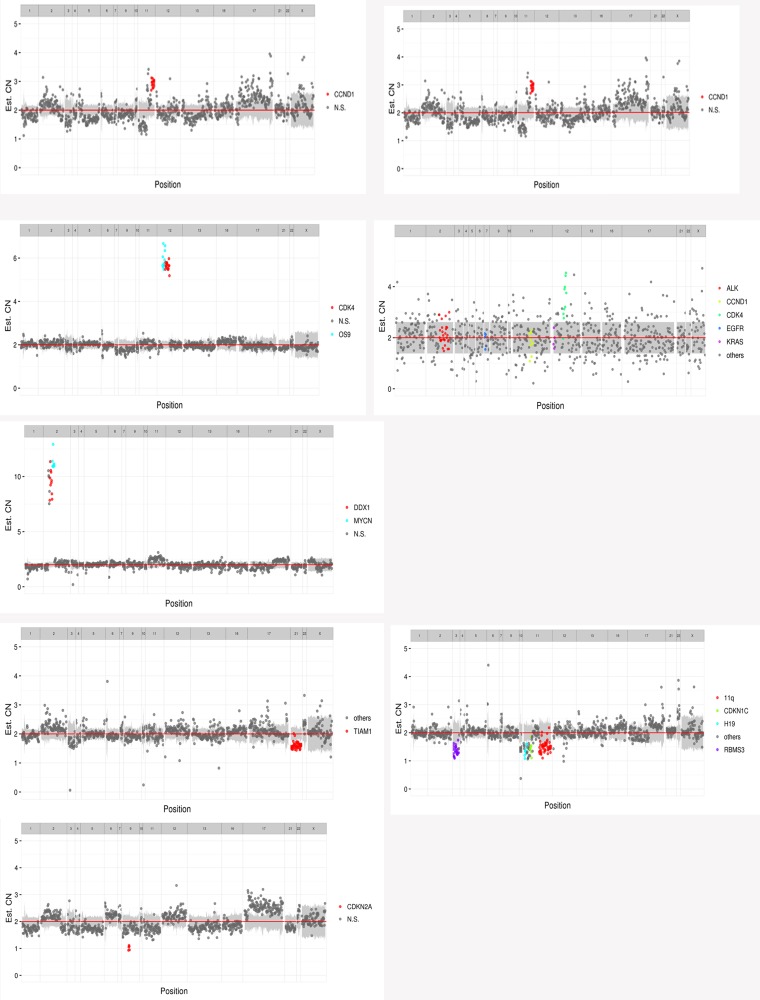
13 CNVs of 8 patients (CN_amp: copy number amplification; CN_del: copy number deletion)

The 33 biopsies and matched leukocyte cells were subjected to capture-based targeted sequencing. With the obtained high-depth sequencing data, the mutations in all of the cases were accurately quantified. Overall, 17 unique somatic aberrations, including 14 genes, were identified in 11 patients, and 13 CNVs were detected in 8 patients: 2 amplifications were observed in *CDK4* of 12q14 and *CCND1* of 11q13, 1 amplification was found in *OS*9 of 12q13, *MYCN*, and *DDX1* of 2p24, and 1 deletion was located in 11q, cyclin-dependent kinase inhibitor 1C (*CDKN1C*) of 11p15.4, *CDKN2A* of 9p21, *H19* of 11p15.5, *RBMS3* of 3p24.1, and T-cell lymphoma invasion and metastasis 1 (*TIAM1*) of 21q22.11 (Figure 1).

**Figure 2:**
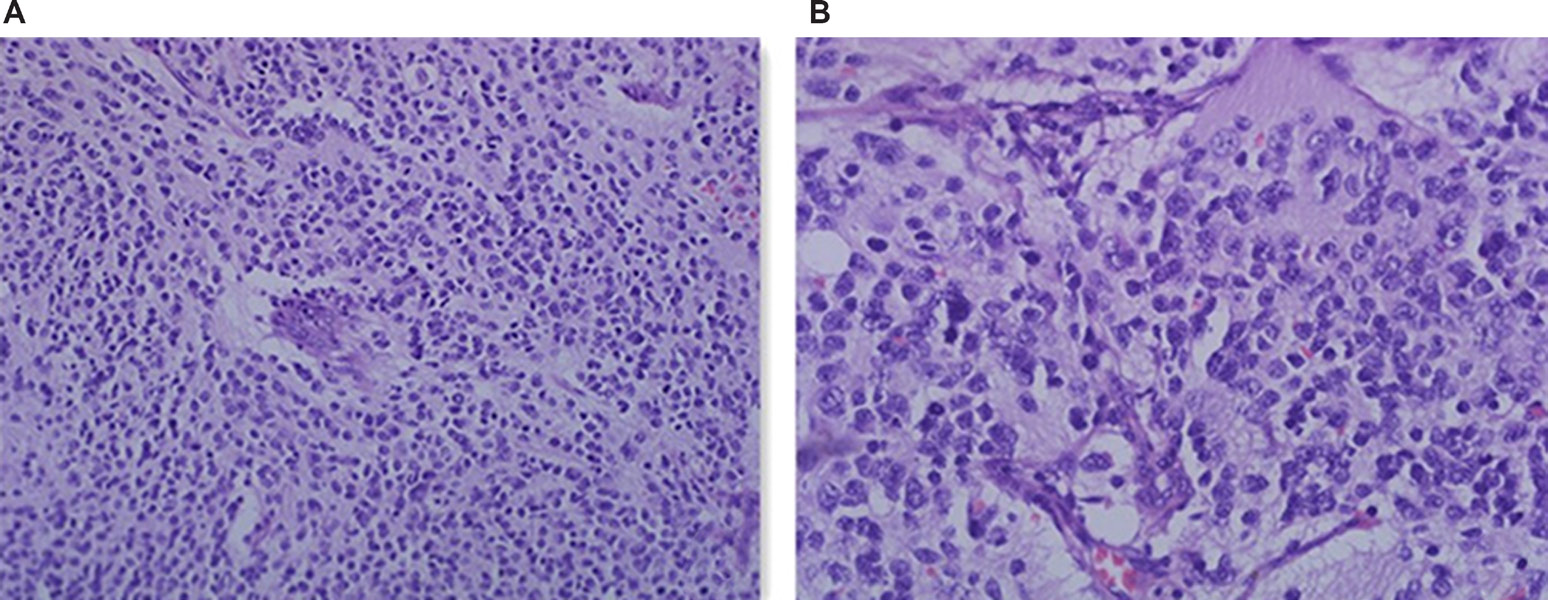
H&E staining of the patient with *TIAM1* deletion **(A)** HE 200 ×; **(B)** HE 400 ×

TIAM1 gene deletion in one patient was a newly found variation, and the differentiated degree of neuroblast cells was poor. The reviewed pathological hematoxylin and eosin (H&E) staining results of the flat pieces of the tumor sample in microscopic 200 × and 400 × forms are shown in Figure 2.

**Figure 3:**
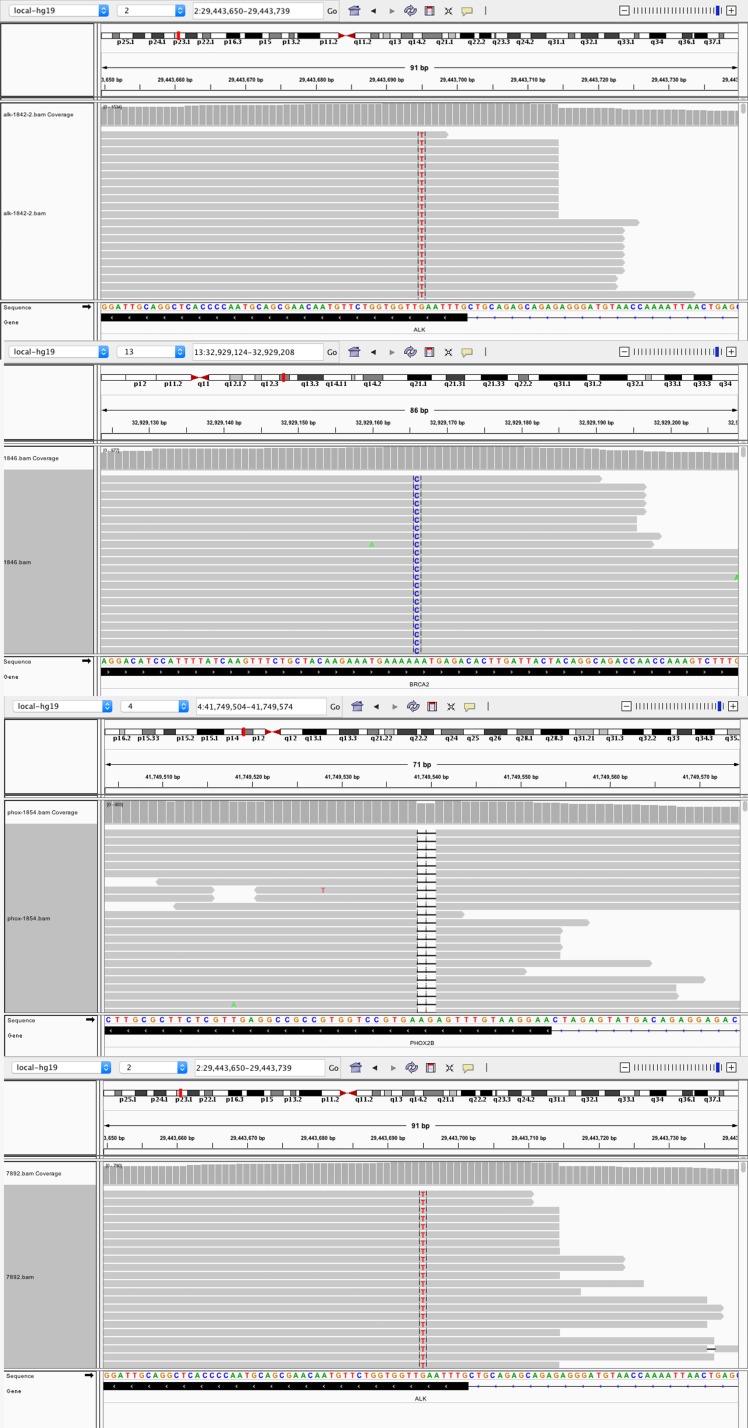
Four point mutations in four patients (2 ALK: F1174L; BRCA2: K2392N; *PHOX2B*: F86fs)

In addition to CNVs, three missense variants were identified in three patients (*ALK*:2*F1174L, *BRCA2*: K2392N) and one frameshift variant was detected in one patient (*PHOX2B*: F86fs) in this cohort (Figure 3). *ALK* was previously reported as a frequently mutated gene in NB cases in terms of somatic mutations. All of the *ALK* mutations occurred in the kinase domain, and this observation suggested their pathogenic nature.

**Figure 4:**
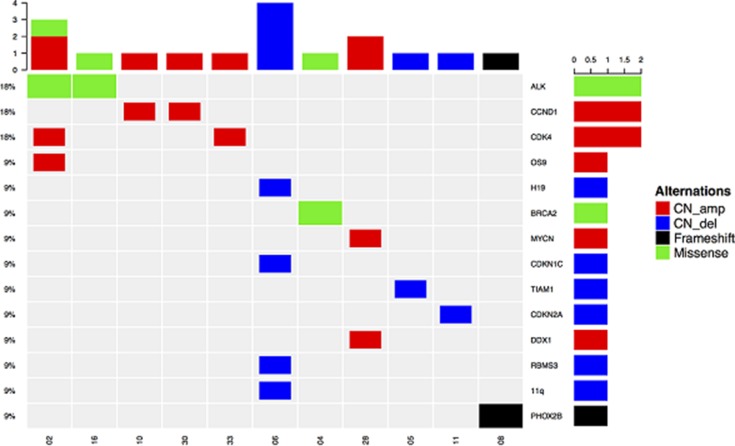
Oncoprint outcome of 11 patients (detected somatic mutations were mostly CNVs, including CN amp and CN del) *CDK4*, *CCND1*, and *ALK* variations appeared most frequently, and they were present in two patients)

All of the changes, including CNVs, accounted for 76.5% (13/17) in comparison to 23.5% (4/17) of point mutations were detected at a high frequency. The results of somatic mutations are illustrated in Figure 4 and Supplementary Table 4.

### Identification of CNVs associated with other prognostic factors

In this study, 11 CNVs among 32 patients, in which one case of amplification of *MYCN* and *DDX1* was excluded, were associated with other significant prognostic effects, namely, age at diagnosis, INSS, INPC, and distant metastasis. In Table 2, CNVs were also associated with UH (*P* = 0.003) and more likely to occur in stage 4 (*P* = 0.041). Only one deletion occurred in *TIAM1* in stage 1. All amplifications occurred in patients in stage 4 and in the UH group.

**Table 2:** Clinical and tumor characteristics of the discovery cohort (*n* = 32)

Characteristic	Number (%)	CNV (%)	*P*-value
Age			0.465
≤ 18 months	12 (37.5)	2 (6.3)	
> 18 months	20 (62.5)	5 (15.6)	
Gender			0.500
boy	16 (50)	3 (9.4)	
girl	16 (50)	4 (12.5)	
INSS			**0.041**
non–stage 4	16 (50)	1 (3.1)	
4	16 (50)	6 (18.8)	
Histology (INPC)			**0.003**
Favorable (FH)	16 (50)	0 (0)	
Unfavorable (UH)	16 (50)	7 (21.9)	
Distant Metastatic			0.061
+	17 (53.1)	6 (18.8)	
−	15 (46.9)	1 (3.1)	
Serum NSE			0.389
> 100 ng/mL	19(59.4)	5 (15.6)	
≤ 100 ng/mL	13(39.4)	2 (6.3)	

### Identification of CNVs associated with prognosis

**Figure 5:**
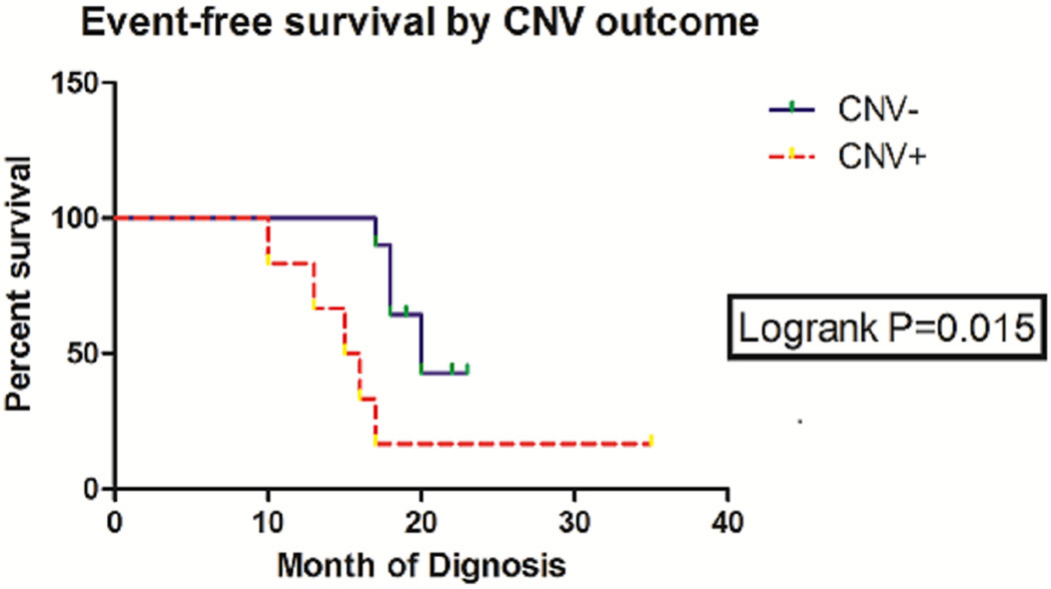
Summary of three-year EFS rates and log-rank test comparisons by CNV outcome

16 patients in Stage 4 received the same chemotherapy and surgery, lacked MNA, and belonged to nearly the same age group and INPC group to avoid the interference of other prognostic factors. The three-year EFS of patients without CNV (10 patients) was 60.0% in comparison to the EFS (16.7%) of patients with CNV (6 patients) (HR = 0.1344; 95%, CI = 0.027 to 0.678; *P* = 0.015, log-rank test; Figure 5). Of the 16 patients, 9 experienced adverse events: 1 patient died, 2 patients underwent relapse, and 6 patients exhibited progression. Of the 9 patients, 7 manifested definite somatic events, mostly CNVs (Table 1).

### Identification of CNVs associated with treatment effects

**Figure 6:**
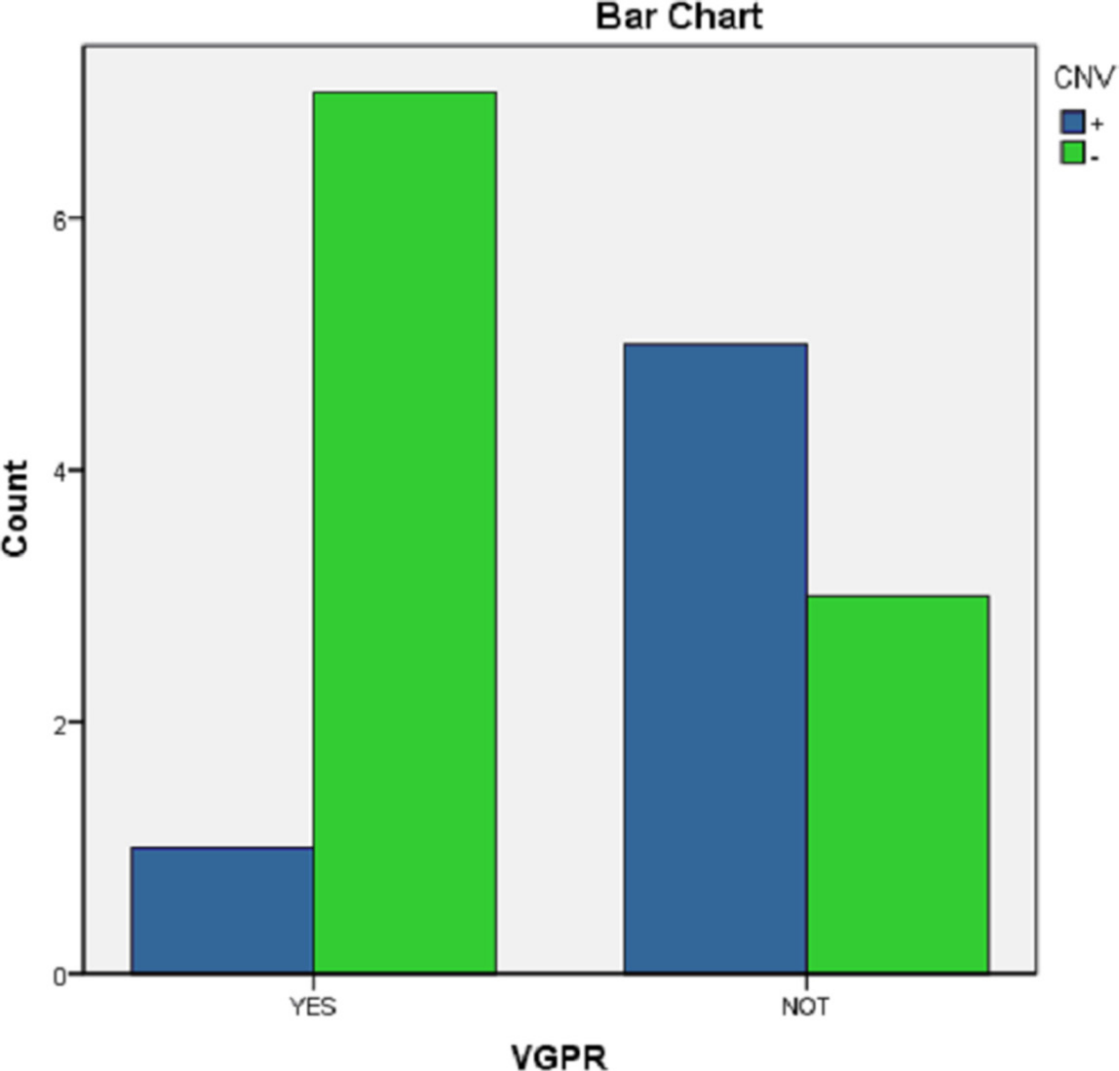
Distribution of obtaining a VGPR after four cycles of chemotherapy between CNV(+) and CNV (−)

The differences between CNV(+) and CNV(−) of the 16 patients were analyzed in terms of their sensitivity to chemotherapy and dependence on very good partial response (VGPR) after four cycles of chemotherapy. The results showed that VGPR in the patients with CNVs were generally difficult to achieve (*P* = 0.059; Figure 6 and Table 1).

### Value of four point mutations

Three missense variants were found in three patients (*ALK*:2*F1174L, *BRCA2*: K2392N) and one frameshift variant in one patient (*PHOX2B*: F86fs) in this cohort, and these conditions accounted for 23.5% of all the results. *BRCA2* was found in the patients with stage 1 and in the FH group. Other point mutations were observed in the patients with stage 4 and in the UH group. The patients with *ALK* or *PHOX2B* progressed eventually.

## DISCUSSION

The amplification of *MYCN* gene (MNA) or the v-myc avian myelocytomatosis viral oncogene NB-derived homolog is a strong independent adverse prognostic factor associated with other adverse prognostic factors. For example, MNA is related to UH, old age, and INSS stage 4 [7, 11]. MNA is also found in a high proportion of adrenal primary tumors [22], and its incidence may differ according to the involvement of specific metastatic sites [23]. However, most patients lack MNA [13], and their prognosis remains poor. A comprehensive and definitive analysis of clinical and other genetic predictors in a large cohort of patients without MNA has yet to be performed. Most amplifications, such as *ALK*, *DDX1*, and *ODC1*, often occur concomitantly with MNA. Other CNVs, such as the amplifications of *CDK4*, *CCND1*, and *CDK6* and deletions of *CDKN2A* and *RBMS3*, which are nonsyntenic with the *MYCN* locus, likely yield a low recurrence to the amplicon of *MYCN*, but these findings have yet to be investigated [24–29]. However, the frequency of recurrent CNVs is relatively higher than that of point mutations [19, 20], which are essential for patients without MNA.

To investigate the significance of other CNVs in these samples without MNA and screen other mutations, we applied capture-based NGS, integrated key CNV and point mutation regions related to NB, and deeply sequenced 33 patients without MNA defined by FISH. Our NB panel comprised 53 genes and 3 large chromosome regions. All of the 33 tissue and leukocyte samples sequenced in this study were subjected to stringent QC allegations. A mean coverage depth of 887× was found in all of the target regions in all of the samples. The mapped read percentage was over 99%. Finally, 13 CNVs were detected in 33 patients and 4 significant point mutations were screened. The reliability of our approach was validated, and this approach was compared with traditional CNV detection method (FISH) in terms of MNA evaluation. Our results indicated that specificity, negative predictive value, and false-positive rate were 97%, 100%, and 3%, respectively. Furthermore, 1 case of MNA was detected through NGS possibly because this method could capture smaller amplified fragments.

Our sequencing results revealed that CNVs were commonly detected. These CNVs were related to other prognostic factors, and they were detected mostly in patients in stage 4 and UH group. These CNVs could also suggest poor prognosis in stage 4 patients who received the same treatment regimen and presented poor responses to conventional chemotherapy. These CNVs showed important clinical values in the predicting prognosis and guiding the targeted therapy of NB in patients without MNA. Our conclusion is consistent with previous findings, which revealed that somatic chromosomal imbalance variation is an important feature of the poor prognosis of NB. For example, the deletions of chromosomes 1p, 3p, 4p, and 11q, the gain of chromosomes 1q, 2p, and 17q, and *MYCN* amplifications were associated with aggressive clinical features, and this finding implied poor prognosis [10, 16–18]. The LOH of chromosome 1p36 and the gain of 17q in NB were closely related to other high-risk clinical features, such as old age, *MYCN* amplification, and distant metastasis [10, 30–33]. In the presence of *MYCN*-amplified NB, the deletion fragment of 1p is often larger [25, 27]. Furthermore, 11q deletion is frequently found in tumors without *MYCN* and associated with poor prognosis in NB [10]. In our study, three large chromosomal regions captured in our assay panel were selected. Finally, 1 case of 11q deletion was found in our study, and the patient developed disease progression after 16 months, and this finding fully confirmed that 11q deletion could be a poor prognostic indicator of NB. Although the relationship between these chromosomal fragments and NB has been clarified, the CNV values in NB clinical treatment, prognostic analysis, and pathogenetic studies remain unclear.

Our study on CNVs found that *CDK4* and *CCND1* amplifications were the most common, and 11q, *CDKN2A*, and *RBMS3* deletions have been detected in NB [24–29, 31, 34, 35]. Other CNVs, such as deletions of *H19* [36] and *CDKN1C* [37], have been rarely reported. 11q deletions and other CNVs also occur predominantly in tumors without MNA, and this observation is consistent with that in previous studies [27, 28]. *CDKN2A* encodes p16INK4a transcripts and functions as an inhibitor of the cell cycle activators *CDK4* and *CDK6*, whose activity is required for cell cycle G1/S transition [38]. *CCND1* or cyclin D1 forms a complex and serves as a regulatory subunit of *CDK4* or *CDK6. OS-9* gene, an endoplasmic reticulum lectin, is usually co-amplified with *CDK4* [39]. *H19*, which is imprinted and maternally expressed transcript and non-protein coding, and *CDKN1C* in 11p15.5 regions are two imprinted genes implicated in cell cycle and associated with Beckwith–Wiedemann syndrome, which is a rare pediatric overgrowth disorder [40]. Most of these genes are involved in the cell cycle, and this finding suggests that NB may be susceptible to being addicted to individually activated oncogenes during cell cycle regulation. Cell cycle-related molecular inhibitors may be used to treat NB. For example, CDK4/6 inhibition has provided an effective strategy to treat a subset of NB tumors [41].

Among the 17 mutations observed in this study, *TIAM1* deletion is a new finding. This gene encodes the protein of the GEF family members and activates the downstream Rac1 protein [42]. Once the neurotrophic factor NGF binds to the neurotrophin receptor TrkA, it activates TIAM1 through Ras and then converts Rac1-GDP into a biologically active Rac1-GTP to act on actin and promote neuronal outgrowth and Schwann cell migration [43]. By contrast, P75NGFR/RhoA pathway functions as a negative regulatory TrkA/TIAM1/Rac1 pathway and thus facilitates the outward retraction of protruding axons and the conversion of cells to become round [44, 45]. The balance of these signaling pathways regulate the growth of neuronal axons and the migration of Schwann cells, which is the process of nerve cell differentiation [46, 47]. NB is derived from primordial neural crest cells, and other immature neuronal cells, such as neuroblasts and gangminic neuroblast cells, are produced because of the differentiation disorder of neural stem cells into ganglion cells and Schwann cell and causes the occurrence of NB [48]. Hence, NB can be used as a good model to examine the differentiation mechanism of tumors [6]. The exploration of genes and molecules involved in NB differentiation is the key to understanding the pathogenesis of this condition in various genes and signaling pathways. However, the accurate differentiation mechanism has yet to be established because of the complexity of signal pathways and molecules participating in NB differentiation. Ras/MAPK signaling pathway is primarily involved in NB differentiation and mediated by neurotrophic factor receptor (NGFR) [49–51]. However, this signaling pathway is implicated in the occurrence and development of other adult epithelial tumors, and Ras/TIAM1/Rac1 pathway likely participates in this mechanism. The Trk/TIAM1/Rac1 pathway promotes the role of positive differentiation. P75NGFR/RhoA negatively regulates this process and participates in dedifferentiation. Therefore, the two signaling pathways modulate NB differentiation. *TIAM1* deletion causes the dedifferentiation of nerve cells and thus leads to tumor occurrence. Molenaar JJ et al [52]. reported that three cases of *TIAM1* inactive mutations are detected in 87 patients with NBs and the downstream signaling pathway is blocked, and this finding is consistent with our results. Either *TIAM1* mutation or deletion blocks the Trk/TIAM1/Rac1 pathway, and this finding indicates the importance of gene and signaling pathway. To verify this observation, we reviewed the diagnosis of patients with *TIAM1* deletion and to observe the morphological characteristics of tumor cells microscopically. Our results revealed that the differentiation of neural cells was poor, and the diagnosis of children was in the UH group. Through the NGS of the NB patients, *TIAM1* was screened more scientifically, and this finding showed a basis for the analysis of NB pathogenesis. Therefore, target-capture NGS is an important tool to screen disease pathogenesis and provided insights into protein functional verification.

This study demonstrated that other CNVs, such as *CDK4* and *CCND1* amplifications and *CDKN2A*, *CDKN1C*, and *RBMS3* deletions, could be the superior choice to identify patient stratification in *MYCN*-negative tumors. These CNVs are associated with NB prognosis and other risk factors. Patients with CNVs usually elicit poor responses to conventional chemotherapy. Comprehensive genetic/genomic profiling through in-depth NGS rather than focusing on single genetic markers could include prognostic information and improve targeted therapy. Further analysis through clinical trials and sample expansion are required to enhance the risk-group stratification in patients without MNA. *TIAM1* deletion is a new mutation that may be involved in NB differentiation and may provide a basis for NB pathogenesis by using NGS screening method.

## MATERIALS AND METHODS

### Patient selection and analysis

The electronic medical records of 127 pediatric patients who underwent diagnostic workup for NB in our medical center between 2012 and 2015 were retrospectively reviewed. Of these patients, 96 suffered from NB and underwent *MYCN* tested by FISH, and these patients were enrolled in this study. Their clinical stages were determined according to the INSS [9] and histologic classification from INPC [7, 53]. Tumor groups were reviewed by the Department of Pathology in Tianjin Medical University Cancer Institute and Hospital. The patients were treated according to international treatment protocols. Distant metastasis refers to the metastasis of tumors in sites that the primary tumor is not connected. Efficacy evaluation and VGPR were determined as follows [54]: primary tumor volume was reduced from 99% to 90%. All of the measurable metastases disappeared, catecholamines and metabolites were recovered to normal levels, and 99Tc scan bone lesions must be positive because bone metastases are not healed, but all of the lesions should be negative in the MIBG examination. MNA was defined by international criteria with FISH [12].

To explore the CNV-guided risk stratification, we enrolled 33 patients from those MNA(−) patients: 16 patients in non-stage 4 were randomly selected, and the 17 other patients were carefully selected(The set of conditions was as follows: (a) stage 4, (b) only received the same chemotherapy and surgery, and (c) age, and the INPC group was as far as possible consistent. This set ruled out the effect of stage, treatment differences, age, and INPC on prognosis). All tumor tissue samples were confirmed by pathology assessment to contain tumor cells more than 60%.

The objects for survival analysis were defined as MNA(−) showed by NGS and Stage 4 of neuroblastoma to avoid the interference of other prognostic factors. Survival was statistically analyzed with SPSS and GraphPad Prism. The EFS event was defined as the first occurrence of relapse, progression, secondary malignancy, or death from any cause. In the absence of an event, the survival time was censored in the last known time. The time to the event was determined from the date of diagnosis. Two-sided log-rank tests were conducted to examine the differences between survival curves. Survival curves were evaluated according to Kaplan–Meier method and compared via a log-rank test with P < 0.05, which was considered significant. Clinical analysis was performed in chi-square test. In all of the analyses, P-values less than 5% were considered significant.

### Tumor tissue collection and DNA extraction

For each patient enrolled in the study, tissue biopsy was obtained from our hospital tissue bank with agreement. DNA was extracted using DNeasy Blood & Tissue Kit according to the manufacturer's instructions. DNA concentration was measured by Qubit dsDNA assay.

### Preparation of white blood cells

At time of biopsy, 10 ml of peripheral blood was obtained, stored in ethylenediaminetetraacetic (EDTA) acid tubes and let it stand at room temperature for 72 hours. The blood cells was stratified by centrifuging for 10 min at 2000 g at 4°C. The the white blood cells was then removed to a new tube and stored at −20°C until further analysis. gDNA was recovered from 4 to 5 ml 200ul of nucleated cell layer using the DNeasy Blood & Tissue Kit (Qiagen). Quantification of gDNA was performed using the Qubit 2.0 Fluorimeter with the dsDNA HS assay kits (Life Technologies, Carlsbad, CA). A minimum of 50 ng of gDNA is required for NGS library construction.

### NGS library preparation

DNA shearing was performed using transposase, synchronized fragmentation and adaptor ligation. Fragments of size 200–400 bp were selected by bead (Agencourt AMPure XP Kit), followed by hybridization with capture probes baits, hybrid selection with magnetic beads and PCR amplification. A bioanalyzer high-sensitivity DNA assay was then performed to assess the quality and size of the fragments and indexed samples were sequenced on Nextseq500 sequencer (Illumina, Inc., USA) with pair-end reads.

### Capture-based targeted DNA sequencing

DNA concentration and genomic DNA quality were measured by Qubit dsDNA assay and 260 nm/280 nm absorption ratio, respectively. DNA was hybridized with the capture probes baits, selected with magnetic beads and PCR amplified. Quality and size range of amplified fragments were then assessed by bioanalyzer high-sensitivity DNA assay, and indexed samples sequenced on a NextSeq 500 sequencer with pair-end reads.

### Sequence data analysis

Sequence data were mapped to the human genome (hg19) using BWA aligner 0.7.10. Local alignment optimization, variant calling and annotation were performed using GATK 3.2, MuTect, and VarScan. Tumor sample was compared against paired white blood cells to identify somatic variants. Variants were filtered using the VarScan fpfilter pipeline, with loci with depth less than 100 filtered out. Minimal of 5 supporting reads were required for INDELs and 8 supporting reads were required for SNV calling. Variants were annotated with ANNOVAR and SnpEff v3.6. Annotation database including the ExAC, 1000 Genomes, dbSNP, ESP6500SI-V2, and COSMIC. DNA translocation analysis was performed using both Tophat2 and Factera 1.4.3. CNV were identified using in-house R scripts based on the coverage ratio of the capture intervals in tumor and normal samples. Coverage depth data were corrected for the sequencing bias due to GC content and probe density. The mean centers and variation levels of coverage depth were then calculated from normal white blood cells as reference. The difference of adjusted coverage depth for each gene from tumor sample and reference was evaluated by *t*-test. Genes with *p*-value < 1e-4 in the *t*-test and CN > 2.5 or < 1.5 were considered as amplification or deletion.

## SUPPLEMENTARY MATERIALS FIGURE AND TABLES




