# Supplementary material for: Clinical evaluation of integrated panel testing by next-generation sequencing for somatic mutations in neuroblastomas with MYCN unamplification
Source: Oncotarget. 2017 May 17;8(30):49689–701. doi: 10.18632/oncotarget.17917 (PMC5564799; doi:10.18632/oncotarget.17917)
Supplement: Supplementary file 1 [file oncotarget-08-49689-s001.pdf]

Clinical evaluation of integrated panel testing by next-generation sequencing for somatic mutations in neuroblastomas with MYCN unamplification

Supplementary Materials

Supplementary Table 1: Clinical data of 33 patients

"Patients NO."	Gender	"Age (month)"	INSS	"Distance metastasis"	INPC	"NSE (ng/mL)"
1	male	16	3	−	FH	38.25
2	female	72	4	+	UH	137.1
3	female	6	3	−	FH	219.5
4	male	13	1	−	FH	28.94
5	female	11	1	−	UH	20.35
6	male	53	4	+	UH	65.39
7	male	36	1	−	FH	44.14
8	male	40	4	+	UH	1282
9	female	11	4s	+	FH	120.8
10	male	27	4	+	UH	595.2
11	male	12	4	+	UH	790.7
12	male	8	3	−	FH	209.2
13	female	11	3	−	FH	79.77
14	female	108	1	−	UH	19.81
15	female	47	4	+	UH	1008
16	female	81	4	+	UH	344.1
17	female	71	4	+	UH	1398
18	male	5	1	−	FH	22.81
19	male	67	4	+	UH	112.2
20	male	57	4	+	FH	502
21	male	72	3	−	FH	224.4
22	male	39	4	+	UH	432.53
23	female	15	1	−	FH	14.86
24	male	38	2	−	FH	36.7
25	female	8	2	−	UH	82.66
26	male	52	4	+	UH	230.9
27	female	53	1	−	FH	12.45
28	female	48	4	+	UH	1185
29	female	15	1	−	FH	23.66
30	female	62	4	+	UH	320.3
31	female	40	4	+	FH	354.4
32	male	42	4	+	FH	240.3
33	female	36	4	+	UH	518.7

Supplementary Table 2: Panel design and NGS data quality assessment

17q	*CDK4*	*MDM2*	*MYCN*	*ALK*	*FGFR4*	*CCND1*
CDK6	*DDX1*	*GLI1*	*NBAS*	*ODC1*	*OS9*	*YEATS4*
11q	*1p36*	*ATRX*	*ARID1A*	*ARID1B*	*CDKN2A*	*CDKN1C*
H19	*IGF2*	*RBMS3*	*PTEN*	*PTPRD*	*PHOX2B*	*LMO1*
APC	*AXIN2*	*BRAF*	*CTNNB1*	*FBXW7*	*HRAS*	*KRAS*
NF1	*NRAS*	*PIK3CA*	*PTPN11*	*TIAM1*	*TP53*	*BARD1*
BCOR	*BRCA1*	*BRCA2*	*CHEK2*	*EGFR*	*ERBB2*	*FANCA*
KIT	*PALB2*	*PDGFRA*	*PINK1*	*PTCH1*	*RB1*	*WT1*

Our panel, mainly CNV-based, covers most of the chromosomal regions and candidate genes which have been reported to associate with neuroblastoma. We developed the panel to select 3 large chromosomal regions(17q, 11q and 1p36) that are most reported and others associated with MYCN amplification or not, including MYCN itself and ALK, DDX, CDK4, CDK6, MDM2, CCND1, FGFR4, GLI1, NBAS, ODC1, OS9, YEATS4, deleted on ATRX, ARID1A, ARID1B, CDKN2A, CDKN1C, H19, IGF2, RBMS3, PTEN, PTPRD, TIAM1 and NF1. In order to expand the screening of somatic mutations of neuroblastoma, the panel we designed also includes important genes with somatic mutations found previously in neuroblastoma, for example, PTPN11, PHOX2B, BARD1, PTCH1, TP53, CHEK2, BRCA1, BRCA2, HRAS, KRAS, NRAS, PIK3CA and BRAF. Overall, our neuroblastoma panel, spans 53 genes and 3 large chromosome regions.
Description: The gray shade is the area that is captured based on the CNVs, and the other is the capture area based on the point mutations.
All of the 33 tissue and leukocyte samples sequenced were then subjected to stringent QC allegations. The mean coverage depth in all of the target regions in all of the samples was 887×, and the mapped read percentage was over 99%. The imputed insert size and library complexity statistics shown in Supplementary Figure 1 revealed a mean insert size of 212 bp, and these findings indicated a high capture efficiency of the probes.

Supplementary Table 3: The detailed quality control data of 33 samples.
See Supplementary_Table_3.

Supplementary Table 4: List of the 17 high-confidence somatic mutations identified among 33 patients

Patient NO.	Gene	Mutation_ Type	Exon Rank	Description	AF	CHROM	Reference
02	*ALK*	missense_ variant	23	p.F1174L	6.50%	2p23.2-p23.1	[1]
	CDK4	cn_amp	NA	cn_amp	6.69	12q14.1	[2]
	OS9	cn_amp	NA	cn_amp	5.13	12q13.3	[3]
04	*BRCA2*	missense_ variant	14	p.K2392N	4.89%	13q13.1	[4, 5]
05	*TIAM1*	cn_del	NA	cn_del	1.5	21q22.11	***NEW***
06	*11q*	cn_del	NA	cn_del	1.21	11q	[6]
	CDKN1C	cn_del	NA	cn_del	1.32	11p15.4	[2]
	H19	cn_del	NA	cn_del	1.23	11p15.5	[7, 8]
	RBMS3	cn_del	NA	cn_del	1.19	3p24.1	[9]
08	*PHOX2B*	frameshift_ variant	2	p.F86fs	9.10%	4p13	[10]
16	*ALK*	missense_ variant	23	p.F1174L	16.20%	2p23.2-p23.1	[1]
28	*DDX1*	cn_amp	NA	cn_amp	10.05	2p24.3	[11]
	MYCN	cn_amp	NA	cn_amp	10.52	2p24.3	[11]
33	*CDK4*	cn_amp	NA	cn_amp	3.46	12q14.1	[2]
10	*CCND1*	cn_amp	NA	cn_amp	2.93	11q13.3	[2]
11	*CDKN2A*	cn_del	NA	cn_del	1.02	9p21.3	[2, 9]
30	*CCND1*	cn_amp	NA	cn_amp	2.96	11q13.3	[2]

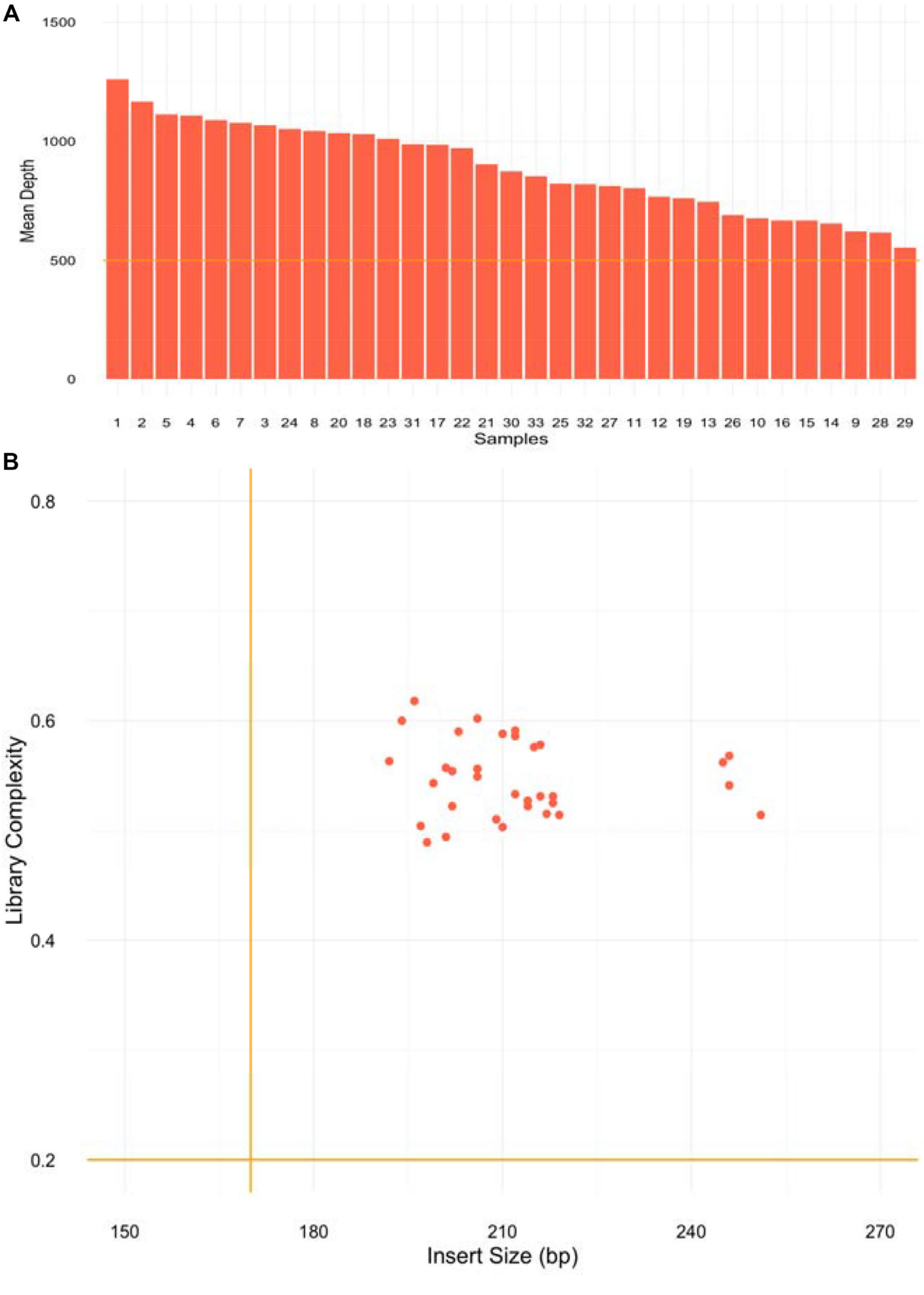

Supplementary Figure 1: Quality assessment of the targeted sequencing data among 33 samples. (A) The mean sequencing depth of 33 samples(the mean coverage depth was well distributed among regions, with a minimum of 554× and a maximum of 1259×); (B) The insert size and Library complexity of 33 samples (The insert size means insertion length, to measure the degree of DNA degradation, more than 170 is qualified. Library complexity means the complexity of the library, used to measure the number of the original DNA template, more than 0.2 is qualified. Insert size and covered library complexity of our 33 samples were qualified).

REFERENCES

1. Berry T, Luther W, Bhatnagar N, Jamin Y, Poon E, Sanda T, Pei D, Sharma B, Vetharoy WR, Hallsworth A, Ahmad Z, Barker K, Moreau L, et al. The ALK(F1174L) mutation potentiates the oncogenic activity of MYCN in neuroblastoma. Cancer cell. 2012; 22:117–130.
2. Molenaar JJ, Koster J, Ebus ME, van Sluis P, Westerhout EM, de Preter K, Gisselsson D, Ora I, Speleman F, Caron HN, Versteeg R. Copy number defects of G1-cell cycle genes in neuroblastoma are frequent and correlate with high expression of E2F target genes and a poor prognosis. Genes, chromosomes & cancer. 2012; 51:10–19.
3. Liu C, Li D, Hu J, Jiang J, Zhang W, Chen Y, Cui X, Qi Y, Zou H, Zhang W, Li F. Chromosomal and genetic imbalances in Chinese patients with rhabdomyosarcoma detected by high-resolution array comparative genomic hybridization. International journal of clinical and experimental pathology. 2014; 7:690–698.
4. Loizidou MA, Hadjisavvas A, Tanteles GA, Spanou-Aristidou E, Kyriacou K, Christophidou-Anastasiadou V. Fanconi anemia-D1 due to homozygosity for the BRCA2 gene Cypriot founder mutation: A case report. Oncology letters. 2016; 11:471–473.
5. Malric A, Defachelles AS, Leblanc T, Lescoeur B, Lacour B, Peuchmaur M, Maurage CA, Pierron G, Guillemot D, d'Enghien CD, Soulier J, Stoppa-Lyonnet D, Bourdeaut F. Fanconi anemia and solid malignancies in childhood: a national retrospective study. Pediatric blood & cancer. 2015; 62:463–470.
6. Sridhar S, Al-Moallem B, Kamal H, Terrile M, Stallings RL. New insights into the genetics of neuroblastoma. Molecular diagnosis & therapy. 2013; 17:63–69.
7. Serra A, Eirich K, Winkler AK, Mrasek K, Gohring G, Barbi G, Cario H, Schlegelberger B, Pokora B, Liehr T, Leriche C, Henne-Bruns D, Barth TF, et al. Shared Copy Number Variation in Simultaneous Nephroblastoma and Neuroblastoma due to Fanconi Anemia. Molecular syndromology. 2012; 3:120–130.
8. Wada M, Seeger RC, Mizoguchi H, Koeffler HP. Maintenance of normal imprinting of H19 and IGF2 genes in neuroblastoma. Cancer research. 1995; 55:3386–3388.
9. Caren H, Erichsen J, Olsson L, Enerback C, Sjoberg RM, Abrahamsson J, Kogner P, Martinsson T. High-resolution array copy number analyses for detection of deletion, gain, amplification and copy-neutral LOH in primary neuroblastoma tumors: four cases of homozygous deletions of the CDKN2A gene. BMC genomics. 2008; 9:353.
10. Normand C, Michon J, Janoueix-Lerosey I, Delattre O, Schleiermacher G. Genetic alterations in neuroblastoma and their usefulness for clinical management. Bulletin du cancer. 2011; 98:477–488.
11. Defferrari R, Tonini GP, Conte M, Papio F, Sementa AR, Valent A, Schena F, Perri P, Mazzocco K. Concomitant DDX1 and MYCN gain in neuroblastoma. Cancer letters. 2007; 256:56–63.
